# Progress in Genito-Urinary Surgery

**Published:** 1903-06-20

**Authors:** 


### Progress in Genito-Urinary Surgery.

(*Concluded from page* 170.)

**Rupture of the Kidney.**—G. E. Dodge (New York) has written on this subject and reported nine cases. Under the term rupture of the kidney are included contusions without rupture of the capsule and all grades of more complete rupture. The rupture may involve the larger blood-vessels, the pelvis of the kidney, or the ureter. Owing to the structure of the organ the split is commonly transverse, but may take any direction, or the kidney may be torn loose from its pedicle. The hæmorrhage into the cellular tissue may be very free. If the peritoneum is ruptured the tendency is towards uninterrupted hæmorrhage. Laceration of the peritoneum occurs most frequently in children, owing to absence of sub-peritoneal fat. Infection of the extravasated products may result in diffuse infection of the kidney or in the formation of an abscess within or adjacent to the kidney. Urethritis or cystitis, or decomposing blood in the bladder, may originate the infection. The uninjured kidney may become infected secondarily through its ureter. The source of infection may, however, be in some other part of the body, *e g.* in a carbuncle. Where the pelvis of the kidney has been torn there may be extensive extravasation of urine with the formation of a large retroperitoneal cyst. The term pseudo hydro nephrosis is more suitable for this condition than hydronephrosis. The most characteristic symptoms are pain and hæmaturia. It is rare for clots of blood to collect in the bladder and the hæmaturia may be intermittent, late in appearing, or in rare cases absent. Oliguria is common, anuria occasional. The latter is usually due to shock and secretion is usually resumed in from 24 to 48 hours, but if the kidney of the opposite side is absent, injured or diseased, the condition may persist to a fatal termination. A temperature of 100° to 101° Fahr., is often present in the first few days. Considerably higher temperatures are sometimes exhibited without further cause than local tissue reaction. The presence of a tumour-mass in the loin is often difficult to recognise in the early stages, but rigidity of the muscles of the back and flank is an early and characteristic sign. In uncomplicated cases the course is usually a mild one, and the patient is convalescent in from a fortnight to a month. Hæmaturia alone and pain alone sometimes persist for from six weeks to several months Primary and secondary or prolonged hæmorrhage are the most frequent causes of death, and then come septic processes and suppression of urine. The occurrence of a non-septic traumatic nephritis is rare. The treatment for the less severe cases is non-operative. It comprises complete rest, milk diet, the application of an ice-bag to the loin, and morphia for pain. Ergot, gallic acid, and lead acetate may be administered to control the hæmorrhage. After 48 hours, the conditions demanding an incision are continued hæmorrhage, persistent anuria, septic symptoms, and a well-defined tumour-mass. The removal of a large mass of blood and urine, even if not septic, is advantageous as a preventive of the development of septic processes. The kidney itself may require suture, packing, partial nephrectomy, or complete nephrectomy, according to the extent of the injury and the severity of the symptoms. Complete nephrectomy naturally will most effectually check both bleeding and the development of septic processes.

**Nephrectomy by Lateral Extra-peritoneal Incision.**—Peter Paterson [6] discusses the advantages of this mode of removing the kidney and records a successful case. The operation was performed four and a-half years ago for a tumour in the lower pole of the left kidney, which proved to be a carcinoma undergoing colloid change. The incision extended from the left tenth costal cartilage to a point an inch internal to the anterior superior iliac spine. The peritoneum was exposed and "reflected from the abdominal walls till the whole of the anterior surface of the kidney and a considerable part of the renal vessels were exposed." The gland was then removed, a small opening for drainage made in the loin, and the anterior wound closed by suturing in layers. After a month the patient developed a severe dermatitis and mental confusion, but he recovered from both in a few weeks. When seen four and a half years later there was no weakness of the cicatrix, though the man was regularly employed as an engine-fitter. The advantages of operating through this incision are that the kidney is easily accessible, there is no stretching or tension on the vessels, large tumours can be removed, and the ureter can be exposed almost down to the bladder if necessary. If the abdominal cavity has to be opened, owing to adhesions, the operation is no more difficult than that by the abdominal route. The disadvantages are that the opposite kidney cannot be examined through the wound and that there is a risk of ventral hernia. The latter risk is small if the wound runs an aseptic course.

**Complete Excision of the Bladder.**—Herbert Lund[7] records an example of this operation. The patient was a man aged 57 years. He had been ill for about 12 months, had lost much flesh, and was extremely anæmic. The urine contained pus and blood, and was alkaline. Blood was passed at the end of micturition with pain. No induration of the base of the bladder was felt per rectum nor enlargement of the prostate. Supra-pubic cystotomy was first performed, and digital examination revealed a growth of a papillomatous nature chiefly confined to the trigone and lower half of the bladder. It was then decided to remove the bladder. The peritoneum was stripped off the fundus, and this was found to be a tedious procedure. Then the sides of the bladder were freed, numerous fibrous attachments being clamped and ligatured. The ureters were exposed alternately and divided near the bladder. Then the viscus was turned downwards and forwards until the upper border of the prostate came into view. The neck of the bladder was ligatured near the triangular ligament and the organ removed. The hæmorrhage was never great. A pair of sinus forceps was then made to puncture the rectum about four inches above the anus, and through the opening the ureters were drawn down so that about an inch of each lay free in the rectum. Catgut loops previously fixed in each ureter were then drawn out through the anus and tied round a piece of rubber tubing. The ureters were not fixed to the rectum in any other way. The patient died on the third day from uræmia. Urine containing pus had passed freely through the rectum and anus after a tube had been placed in the latter. At the necropsy both ureters and kidneys were found dilated and containing pus. The ureters were adhering well to the bowel, and probably, if the kidneys had been healthy, recovery would have ensued. The first successful operation of complete removal of the bladder was performed by Paulich, of Prague. The patient was a woman, and the ureters were transplanted into the vagina. Two and a half years later the patient remained fairly comfortable. Tuffier and Dujarier recorded the first successful case in a man. In that case the ureters were fixed in the rectum.

[5] Ann. of Surg., Dec., 1902, p. 899. [6] Lancet, March 14, 1903, p. 729. [7] Lancet, Dec., 1902, p. 1624.

